# LincRNA‐p21 alleviates atherosclerosis progression through regulating the miR‐221/SIRT1/Pcsk9 axis

**DOI:** 10.1111/jcmm.16771

**Published:** 2021-09-19

**Authors:** Haojie Wang, Fei He, Bing Liang, Yuanhu Jing, Pei Zhang, Weichao Liu, Bowen Zhu, Dongmei Dou

**Affiliations:** ^1^ Thoracic & Cardiovascular Surgery Huaihe Hospital of Henan University Kaifeng China; ^2^ School of Clinical Medicine Henan University Kaifeng China; ^3^ Institute of Chronic Disease Risks Assessment Henan University Kaifeng China

**Keywords:** atherosclerosis, long intergenic noncoding RNA‐p21, microRNA‐221, proprotein convertase subtilisin/kexin type 9, sirtuin 1

## Abstract

Atherosclerosis (AS) is the main aetiology of coronary heart disease, cerebral infarction and peripheral vascular disease in humans. Long‐noncoding RNA (LincRNA)‐p21 has been reported to participate in the development of AS. Therefore, this study was designed to investigate the mechanism of LincRNA‐p21 on suppressing the development of AS. We fed ApoE^−/−^ mice with a high‐fat diet to induce an AS mouse model where the lesion area of AS and the extent of lipid deposition were measured. The binding of LincRNA‐p21 and miR‐221 or miR‐221 and SIRT1 was measured using a dual luciferase reporter gene assay and RIP. Following loss‐ and gain‐ function assays, CCK8, EdU, Transwell assay and scratch test were performed to determine the biological processes of human aortic endothelial cells (HAECs). miR‐221 was highly expressed while SIRT1 was poorly expressed in AS. LincRNA‐p21 acted as a sponge for miR‐221. miR‐221 targeted and negatively regulated the expression of SIRT1. LincRNA‐p21 promoted the deacetylation of Pcsk9 by SIRT1 by competitively binding to miR‐221, whereby promoting HAEC proliferation, migration and tube formation. In conclusion, LincRNA‐p21 acted as a molecular sponge for miR‐221 to promote deacetylation of the promoter region of Pcsk9 by SIRT1, therefore preventing the development of AS.

## 1 INTRODUCTION

Atherosclerosis (AS) is an arterial disease process that is characterized by focal intravascular aggregation of lipoproteins containing apolipoprotein B, immune and vascular wall cells and extracellular matrix.^1^ AS is the basis of many high mortality rates of cardiovascular diseases worldwide,^2^ which is induced by accumulation of lipids in the walls of the blood vessels. As a natural component of blood vessels, human aortic endothelial cells (HAECs) play a key role in maintaining the integrity of the cardiovascular system by serving as the barrier between the blood and the vascular wall, and HAEC damage is a key factor in the onset of AS.^3^ Oxidized low‐density lipoprotein (LDL) could activate HAECs, which exert pivotal function on the onset and development of AS.^4^ Understanding the molecular mechanisms driving the phenotypic regulation of HAECs is therefore critical for the better treatment of AS.

A previous study has shown that miR‐221 is involved in the development of AS.^5^ miR‐221 is widely distributed in eukaryotes and deeply involved in posttranscriptional regulation of gene expression.^6^ Moreover, long intergenic noncoding RNA (LincRNA) acts as an endogenous RNA sponge for miRNA, regulates the expression of miRNA and interacts with miRNA to mediate the progression of AS.^7^ LincRNA‐p21 is located on chromosome 17, approximately 15 KB upstream of the Cdkn1a (p21) gene.^8^ The relation between LincRNA‐p21 and AS was discussed in previous studies; for example, LincRNA‐p21 is especially poorly expressed in AS plaques of apolipoprotein E‐deficient (ApoE^(−/−)^) mice and LincRNA‐p21 inhibits cell proliferation and induces apoptosis of vascular smooth muscle cells and mouse mononuclear macrophages in vitro.^9^ LincRNA‐p21 acted as a sponge for miR‐221. Next, Sirtuin 1 (SIRT1) was predicted to be a downstream target of miR‐221 through bioinformatics website. The epigenetic regulation of SIRT1 is controlled by miRNA.^10^ SIRT1 and NAD (+)‐dependent histone deacetylase exert significant effects on a variety of biological processes, including lifespan, stress response and cell survival.^11^ SIRT1 is downregulated and involved in the development of AS.^12^, ^13^ Besides, lack of SIRT1 has been linked to an increased expression of the proprotein convertase subtilisin/kexin type 9 (Pcsk9) gene and increased LDL‐cholesterol levels.^14^ Pcsk9 is a secreted protein that promotes low density lipoprotein receptor degradation, hence regulating the plasma level of LDL cholesterol.^15^ Furthermore, Pcsk9 has been revealed to be a gene that regulates and promotes AS.^16^ Therefore, the objective of this study is to investigate the mechanism of LincRNA‐p21 preventing the development of AS through miR‐221/SIRT1/Pcsk9 axis using ox‐LDL‐treated HAECs and AS mouse model.

## 2 MATERIALS AND METHODS

### 2.1 Ethics statement

The study was conducted with the approval of Huaihe Hospital of Henan University in accordance with the *Declaration of Helsinki*, and the patients or donors of the samples have signed written informed consents. Furthermore, all animal experiments were performed in strict accordance with the recommendations in the guide for the care and use of laboratory animals of the National Institutes of Health.

### 2.2 Collection of clinical samples

Peripheral blood samples from 25 patients with AS aged 50.80 ± 6.72 years (AS‐H group, among which 14 males and 11 females were included) and 18 healthy volunteers aged 54.72 ± 6.11 years (Normal‐H group, among which 10 males and 8 females were included) were collected from Huaihe Hospital of Henan University from February 2018 to January 2019.

### 2.3 AS mouse model induction

Sixty specific pathogen‐free male ApoE^−/−^ mice and 15 normal mice (C57BL/6; aged eight‐week and weighting 16‐21 g) were obtained from Beijing University of Medicine Laboratory (Beijing, China). All mice were housed at 18‐23°C with a humidity of 40‐60%. Normal mice (Normal‐M group) were fed with basal diet and ApoE^−/−^ mice (AS‐M group) fed with a high‐fat diet (21% fat and 0.25% cholesterol) for 12 weeks. After that, 15 AS modelled mice set aside as the control, and the remaining 45 modelled mice were injected with lentiviral vectors expressing oe‐LincRNA‐p21, oe‐Pcsk9, or oe‐NC (n = 15). 200 μL of corresponding adenovirus (Fubio Biological Technology Co., Ltd.) was injected into the tail veins of mice once a week (1 × 10^10^ pfu/mL) for 12 weeks, followed with intraperitoneal injection with 3% pentobarbital sodium (P3761; Sigma‐Aldrich) in anaesthetized mice; carotid artery tissues were collected at the last step.

### 2.4 Cell culture

HAECs and HEK293T cells were purchased from American Type Culture Collection (ATCC, USA). All cells were cultured in high glucose DMEM (10569044; Gibco,) supplemented with 10% foetal bovine serum (FBS) (10099141; Gibco), penicillin (100 U/mL) and streptomycin (100 U/mL) (15070063; Gibco) and cultured in a cell incubator containing 5% CO_2_ at 37℃. Part of the cells was cultured with ox‐LDL (50 μg/mL; Beijing Sunshine Biotechnology Co., Ltd.) for 48 hours for further study.

All shRNA, mimic and inhibitors were purchased from Sangon Biotech Co., Ltd. Plasmid construction was completed by the company according to the protocols. HAECs in the exponential growth phase were treated with 0.25% trypsin‐EDTA, passaged and counted (1 × 10^6^ cells/mL). HAECs were then plated into a six‐well plate, and shRNA, mimic or inhibitor were mixed with Lipofectamine 2000 transfection reagent (11668019; Thermo Fisher Scientific), and transfected into the cells when cell confluence reached 60%. After 24 hours of transfection, the cells were collected for further analysis.

The HAECs were treated with inhibitor NC, miR‐221 inhibitor, sh‐NC, sh‐SIRT1‐1, sh‐SIRT1‐2, mimic NC, miR‐221 mimic, oe‐NC, oe‐LincRNA‐p21, or treated with co‐transfection of oe‐LincRNA‐p21 + mimic NC, oe‐LincRNA‐p21 + miR‐221 mimic, oe‐SIRT1, miR‐221 inhibitor +oe‐NC, miR‐221 inhibitor +oe‐Pcsk9. After transfection, cells were treated with ox‐LDL.

### 2.5 Haematoxylin and eosin and Oil red O staining

Haematoxylin and eosin (HE) and Oil red O staining were performed according to the methods mentioned in a study.^17^ The atherosclerotic lesions in mice aortic sinus were observed under the microscope (BX63, Olympus Optical Co., Ltd.).

### 2.6 Reverse transcription quantitative polymerase chain reaction (RT‐qPCR)

Total RNA content of tissues and cells was extracted with TRIzol (16096020; Thermo Fisher Scientific). RNA of miRNA was reversely transcribed using reverse transcription kit TaqMan ™ MicroRNA Reverse Transcription Kit (4366596; Thermo Fisher Scientific) and the RNA of mRNA was reversely transcribed using reverse transcription kit High‐Capacity cDNA Reverse Transcription Kit (4368813; Thermo Fisher Scientific). The primer sequences of lincRNA‐p21, miR‐221, SIRT1, Pcsk9, GAPDH and U6 were designed and provided by Sangon Biotech. The primer sequences are shown in Table S1, with GAPDH as internal reference. Reverse transcription quantitative polymerase chain reaction (RT‐qPCR) kit (11732020; Thermo Fisher Scientific) was used to carry out the RT‐qPCR experiment according to the instructions using $2^{‐\Delta \Delta \text{Ct}}$ method.

### 2.7 Western blot analysis

The total protein content of tissues and cells was extracted by following the instructions provided by the protein extraction kit (78501; Thermo Fisher Scientific). The total protein concentration of each sample was determined using the BCA kit (23229; Thermo Fisher Scientific). The protein sample was then added to each lane with a micro sampler for electrophoretic separation and transferred onto the PVDF membrane (1620177; Bio‐Rad, Hercules, CA, USA). The membranes were blocked in 5% BSA for 1 hour, added with primary and secondary antibodies (Abcam; Table 1), followed by incubation for 1 hour. The membrane was immersed in ECL reaction solution (1705062; Bio‐Rad) for 1 minute and exposed by an ImageQuant LAS 4000 mini imaging system (GE Healthcare). U6 served as the internal reference for miR‐221, while glyceraldehyde‐3‐phosphate dehydrogenase (GAPDH) as the internal reference, the relative expression level of each protein was measured by the ratio of grey value between the target band and the internal reference band.

**TABLE 1:** Antibodies for western blot analysis

Primary or secondary antibody	Antibodies	Catalogue no.	Dilution ratio
Primary antibody	Rabbit polyclonal antibody GAPDH	ab181602	1:5000
Primary antibody	Rabbit polyclonal antibody SIRT1	ab110304	1:1000
Primary antibody	Rabbit polyclonal antibody Pcsk9	ab181142	1:1000
Secondary antibody	Goat anti‐rabbit IgG	ab6721	1:5000

### 2.8 Dual luciferase reporter gene assay

The online analysis website (http://starbase.sysu.edu.cn/) was used to predict targeted binding sites of miR‐221 to SIRT1. Synthesis of miR‐221 and LincRNA‐p21, miR‐221 and SIRT1 gene fragments containing targeted binding (LincRNA‐p21 WT and SIRT1 WT) and mutant fragments (LincRNA‐p21 Mut and SIRT1 Mut) were introduced into the PmirGLO vector (LM1439; LMAI Bio) using T4 DNA ligase (M0204S; New England Biolabs). Renilla fluorescent plasmids and constructed luciferase reporter plasmids were co‐transfected into HEK293T cells with mimic‐NC and miR‐221 mimic. Cells were collected and lysed after 48 hours of routine transfection. Dual luciferase^®^ Reporter Assay System kit (E1910; Promega Corporation) was applied for luciferase activity measurement by using a GloMax^®^ 20/20 Luminometer detector (E5311; Promega Corporation) and relative luciferase activity was determined as previously conducted. All vectors were constructed by Sangon Biotech.

### 2.9 RNA immunoprecipitation

An RNA immunoprecipitation (RIP) kit (17‐701; Millipore) was used to detect the possible binding sites between LincRNA‐p21 and miR‐221 and Ago2 protein in HAECs by following the protocols of the manufacturer. Cells at 80%‐90% confluency were lysed in an ice bath with RIPA lysate (P0013B; Beyotime Institute of Biotechnology) for 5 minutes, and the supernatant was taken after centrifugation at 4°C for 10 minutes at 10 000 *g*. Part of the cell extract was taken as the input, and the remainder was incubated with antibodies for co‐precipitation. The magnetic beads–antibody complex was re‐suspended in 900 μL RIP Wash Buffer, and 100 μL cell extracts were added and incubated overnight at 4°C. The sample was placed on a magnetic base to collect the magnetic bead–protein complex. The samples and input were digested by protease K, and RNA was extracted for subsequent RT‐qPCR. The antibody used for RIP was Ago2 (ab32381; 1:100, Abcam) and was allowed to mix for 30 minutes, and anti‐human IgG (ab182931; 1:100, Abcam) was used as an NC.

### 2.10 Fluorescence in situ hybridization

Fluorescence in situ hybridization (FISH) assay was performed based on the methods mentioned in a study^18^ using the Cy3‐labelled LincRNA‐p21 probe (Guangzhou RiboBio Co., Ltd.) and fluorescein‐5‐isothiocyanate (FITC)‐labelled miR‐221 probe (RiboBio).

### 2.11 Co‐immunoprecipitation

Co‐immunoprecipitation (Co‐IP) assay was performed by following the methods indicated in a study.^19^ The primary antibody SIRT1 (1:200; ab32441, Abcam) and secondary antibodies Pcsk9 (1:50, ab191385, Abcam) and anti‐acetyl Lysine (ab21623, 1:50, Abcam) were used.

### 2.12 Tube formation test

Capillary‐like network formation was performed to detect the angiogenic ability of HAECs. In short, HAECs were inoculated at a density of 2 × 10^4^ cells/well on a 96‐well plate coated with 60 μL Matrigel (BD Bioscience). After 48 hours of culture, the average number of capillary‐like branches was counted in five random microscope fields with a computer‐aided microscope.

### 2.13 Terminal‐deoxynucleotidyl transferase mediated nick end labelling staining

The experiment was conducted according to the operating instructions provided by the apoptosis detection kit (40306ES60/40308ES60, Shanghai Yeasen BioTechnologies Co., Ltd.). The frozen sections were soaked in PBS, and 200 mL of citrate buffer solution (0.1 mol/L, pH6.0) was heated to 90‐95°C and then quickly poured into the sections. The sections were cooled down rapidly, incubated with 20% normal bovine serum for 30 minutes and then with 50 μL of Terminal‐deoxynucleotidyl transferase mediated nick end labelling (TUNEL) reaction mixture at 37°C for 90 minutes (NC samples without the addition of TUNEL reaction mixture). Subsequently, the sections were incubated with 3% H_2_O_2_ in methanol at 37°C for 90 minutes, added with horseradish peroxidase (HRP) solution 50 μL/section and incubated at 37℃ for 30 minutes. Diaminobenzidine/hydrogen peroxide (DAB/H_2_O_2_) was used for colour development, with haematoxylin for lightly staining, followed by dehydration, transparency and mounting. Photographs were taken with an upright fluorescence microscope (BX63, Olympus Optical Co., Ltd). The number of TUNEL positive cells/unit area was taken as the number of apoptotic cells.

### 2.14 Scratch test

Cell motility was assessed by wound healing measurement. Cells were cultured in 6‐well plates (5 × 10^4^ cells/well). At 80‐90% confluency, the monolayer cells were scraped with a sterile 200 μL suction head and cultured under standard conditions for 24 hours. Wound healing was captured at 0 and 24 hours under a phase contrast microscope.

### 2.15 Cell‐counting kit 8 assay

The transfected cells were cultured in a 96‐well plate (1 × 10^4^ cells/well). Next, 10 μL Cell‐counting kit 8 (CCK‐8) solution was added to each well and incubated at 37℃ for 4 hours. The absorbance value at 450 nm was then measured with microplate reader Synergy 4 (BioTek) to indirectly identify the number of living cells.

### 2.16 5‐ethynyl‐2’‐deoxyuridine assay

Cell proliferation was analysed using Cell‐Light 5‐ethynyl‐2’‐deoxyuridine (EdU) Apollo 567 in vitro kit (Guangzhou RiboBio Co., Ltd.). The transfected cells were cultured in a 96‐well plate (1 × 10^4^ cells/well). After 48 hours of culture, 50 μmol L^−1^ of EdU‐labelled medium was added, followed by incubation for an additional 2 hours. The cells were then fixed in 4% paraformaldehyde (PFA), permeabilized in 0.5% Triton×‐100/PBS, and stained with Apollo staining solution and Hoechst33342. Cell images were obtained under an inverted fluorescence microscope IX73‐AIZFL/PH (Olympus Optical Co., Ltd.).

### 2.17 Transwell assay

The cells (1.5 × 10^4^ cells/well, three parallel wells in each group) were resuspended in low serum medium (5% of FBS) and seeded into a 24‐well Transwell plate (Corning) in the apical chamber. The basolateral chamber was filled with complete medium (containing 10% FBS). After 12 hours of incubation, the cells on the upper surface of the filter membrane were washed and cells that have migrated to the lower surface were stained with 0.5% crystal violet. Migration was observed under an optical microscope (Leica DMI6000B).

### 2.18 Statistical analysis

All experimental data were analysed using the SPSS 21.0 statistical software (IBM Corp, Armonk, NY, USA). The measurement data were expressed by the mean ± standard deviation. The two groups of data conforming to the normal distribution were compared using an independent sample *t* test. The data comparisons between multiple groups were performed using one‐way ANOVA, followed by Tukey's post hoc test. Cell activity at multiple time points was analysed using two‐way ANOVA. Pearson correlation analysis was used for analysing miR‐221 and LincRNA‐p21. *P* < .05 indicated that the difference was statistically significant.

## 3 RESULTS

### 3.1 miR‐221 expression is upregulated in AS

**FIGURE 1:**
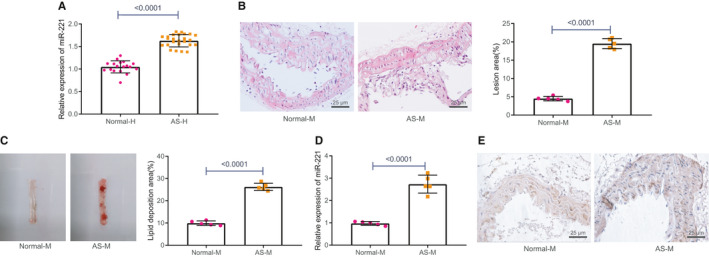
miR‐221 is overly expressed in AS. A, The expression of miR‐221 in peripheral blood of patients with AS (n = 25) and normal patients (n = 18) was measured using RT‐qPCR. B, Arterial damage in mice model of AS was measured using HE staining (×200). C, Lipid deposition area in AS mouse model was detected using Oil red O staining. D, Expression of miR‐221 in tissues of AS mice was tested by RT‐qPCR. E, Expression of miR‐221 in tissues of AS mice was evaluated using FISH (×200). The experiment was repeated three times

The bioinformatics website predicted that miR‐221 was highly expressed in AS. RT‐qPCR displayed that miR‐221 expression was markedly elevated in patients with AS (Figure 1A). Next, AS mouse model was constructed (AS‐M) and HE staining showed that AS mice model exhibited larger lesion area than that in normal mice (Figure 1B). Oil red O staining indicated that the lipid deposition area in AS mice was strikingly increased when compared to that in normal mice (Figure 1C), confirming the successful construction of the AS model. Meanwhile, carotid tissue samples were collected from the mouse model and RT‐qPCR and FISH revealed that miR‐221 expression was notably increased in AS mice model when compared to that in normal mice (Figure 1D, E).

### 3.2 miR‐221 inhibits the proliferation, migration and tube formation of HAECs in vitro

**FIGURE 2:**
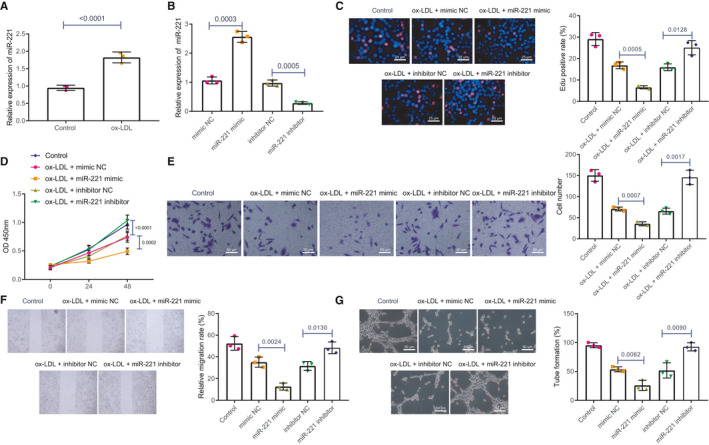
miR‐221 inhibits HAEC proliferation, migration and tube formation in vitro. A, The mRNA expression of miR‐221 in ox‐LDL‐treated HAECs was detected using RT‐qPCR. B, miR‐221 expressions in miR‐221 mimic‐transfected HAECs, miR‐221 inhibitor‐transfected HAECs, inhibitor NC‐transfected HAECs, or mimic NC‐transfected HAECs were measured by means of RT‐qPCR. C, The proliferation ability in HAECs was assessed using EdU assay (×200). D, The proliferation ability in HAECs was detected using CCK‐8. E, The migrative ability in HAECs was measured using Transwell assay (×200). F, Wound healing ability in HAECs was detected using scratch test. G, The angiogenic ability was tested using tube formation test (×200). The experiment was repeated three times

To further identify the biological function of miR‐221 in the phenotypic regulation of HAECs, we performed RT‐qPCR and found that the expression of miR‐221 was significantly increased in ox‐LDL‐treated HAECs (Figure 2A). Next, the HAECs were transfected with miR‐221 mimic and miR‐221 inhibitor and then the transfected HAECs were treated with ox‐LDL. miR‐221 expression in miR‐221 mimic‐transfected HAECs was significantly elevated in comparison with that in mimic NC‐transfected HAECs. However, miR‐221 expression in miR‐221 inhibitor‐transfected HAECs was notably downregulated when compared to that in inhibitor NC‐transfected HAECs, which validated the successful transfection efficiency of miR‐221 in HAECs (Figure 2B). Subsequently, EdU and CCK‐8 assays revealed that overexpressing miR‐221 suppressed ox‐LDL‐treated HAEC proliferation, while inhibiting miR‐221 reversed the inhibitory effect of overexpressing miR‐221 (Figure 2C, D). Transwell assay and scratch test validated that overexpressing miR‐221 inhibited ox‐LDL‐treated cell migration whereas inhibiting miR‐221 improved the decline in HAEC migration owing to the treatment of ox‐LDL (Figure 2E, F). The upregulation of miR‐221 inhibited the ability of ox‐LDL treatment on tube formation in HAECs but silencing miR‐221 promoted the tube formation ability of ox‐LDL‐treated HAECs (Figure 2G).

### 3.3 LincRNA‐p21 acts as a sponge of miR‐221

**FIGURE 3:**
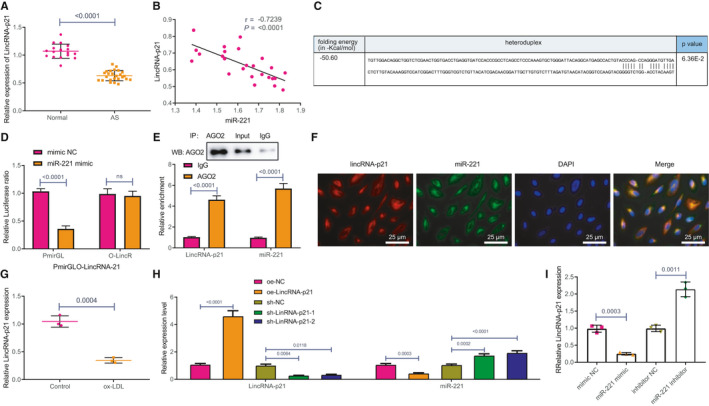
LincRNA‐p21 acts as a molecular sponge for miR‐221. A, LincRNA‐p21 expression in peripheral blood of patients with AS and normal patients was detected using RT‐qPCR. B, The correlation between the expressions of LincRNA‐p21 and miR‐221 in peripheral blood of patients with AS was confirmed by Pearson correlative analysis. C, The binding site between LincRNA‐p21 and miR‐221 was predicted using bioinformatics website. D, The relationship between LincRNA‐p21 and miR‐221 was verified using dual luciferase reporter gene assay. E, Relationship between miR‐221 and LincRNA‐p21 and Ago2 was evaluated using RIP. F, The expression positions of LincRNA‐p21 and miR‐221 in HAECs were analysed by FISH (Red: LincRNA‐p21; Green: miR‐221). G, The effect of ox‐LDL on LincRNA‐p21 in HAECs was observed using RT‐qPCR. H, The expressions of LincRNA‐p21 and miR‐221 after LincRNA‐p21 was overexpressed and inhibited were detected using RT‐qPCR. I, LincRNA‐p21 expression after miR‐221 was overexpressed and silenced was detected using RT‐qPCR. The experiment was repeated three times

Because miR‐221 played key roles in regulating the HAEC phenotype, we investigated the potential regulatory mechanisms of miR‐221 in depth. LncRNAs can act as molecular sponges for endogenous RNAs, interacting with miRNAs and affecting the expression of target genes and interacting with AS.^5^ A study has also shown that LincRNA‐p21 is reduced in patients with coronary heart disease and is potentially involved in the inhibition of AS.^7^ To further explore whether LincRNA‐p21 affected the development of AS through miR‐221, we first detected LincRNA‐p21 in peripheral blood samples of patients with AS, which showed a striking decrease in LincRNA‐p21 expression in the peripheral blood of patients with AS compared with that of normal individuals (Figure 3A). Furthermore, Pearson correlation analysis identified a negative correlation between LincRNA‐p21 and the expression of miR‐221 (Figure 3B). We also predicted the binding site of LincRNA‐p21 and miR‐221 through the bioinformatics website (http://starbase.sysu.edu.cn/) (Figure 3C). The luciferase activity of miR‐221 against LincRNA‐p21 was verified by dual luciferase reporter gene assay. The results showed that overexpressing miR‐221 could significantly inhibit the luciferase activity of the wild‐type binding site of LincRNA‐p21, but there was no effect on luciferase activity at the mutant binding site (Figure 3D). RIP indicated that LincRNA‐p21 and miR‐221 in HAECs were more abundantly expressed in Ago2 precipitates than that in IgG precipitates (Figure 3E). Subsequently, FISH analysis showed that LincRNA‐p21 and miR‐221 co‐localized at the cytoplasm (Figure 3F). In addition, we also found that ox‐LDL treatment inhibited the expression of LincRNA‐p21 in HAECs (Figure 3G). We later found that miR‐221 expression was decreased after LincRNA‐p21 was overexpressed in HAECs, while inhibiting LincRNA‐p21 promoted the expression of miR‐221 (Figure 3H). Furthermore, overexpressing miR‐221 contributed to a downregulated LincRNA‐p21 expression, whereas silencing miR‐221 resulted in a significantly increased LincRNA‐p21 expression (Figure 3I).

### 3.4 LincRNA‐p21 promotes the proliferation, migration and tube formation of HAECs by inhibiting miR‐221 expression

**FIGURE 4:**
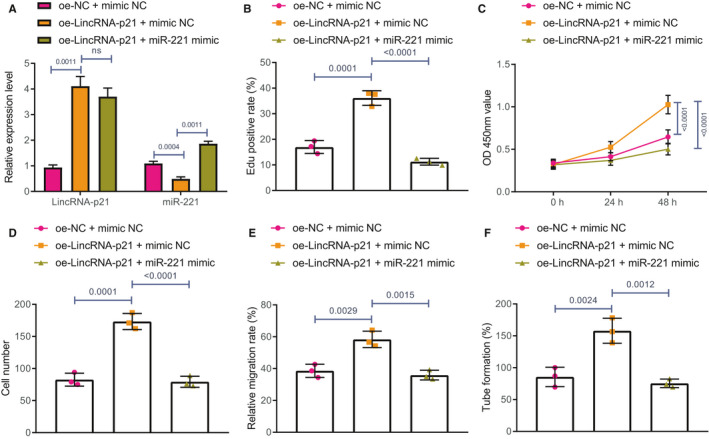
Overexpression of LincRNA‐p21 promotes HAEC proliferation, migration and tube formation through suppressing the expression of miR‐221. HAECs were co‐transfected with oe‐LincRNA‐p21‐1 + mimic NC or oe‐LincRNA‐p21 + miR‐221 mimic exposed to ox‐LDL. A, The expressions of LincRNA‐p21 and miR‐221 in HAECs were detected using RT‐qPCR. B, HAEC proliferation ability was assessed using EdU. C, HAEC proliferation ability was examined using CCK‐8. D, HAEC migrative ability was evaluated using Transwell assay. E, Wound healing ability in HAECs was monitored by the means of scratch test. F, Angiogenic ability in HAECs was tested using tube formation test. The experiment was repeated three times

The results showed that overexpressing LincRNA‐p21 had significantly increased LincRNA‐p21 and decreased miR‐221 expression in ox‐LDL‐treated HAECs. Nevertheless, the inhibitory effect of overexpressing LincRNA‐p21 on miR‐221 would be nullified by the addition of oe‐LincRNA‐p21 + miR‐221 mimic (Figure 4A).

EdU and CCK‐8 assays displayed that overexpressing LincRNA‐p21 promoted the growth of ox‐LDL‐treated HAECs, while overexpressing miR‐221 blocked the promotion of cell growth by LincRNA‐p21 (Figure 4B, C). Transwell assay and scratch test revealed that overexpressing LincRNA‐p21 improved the migrative capabilities of HAECs exposed to ox‐LDL, while overexpressing miR‐221 blocked the promotion of LincRNA‐p21 on cell migrative capabilities in ox‐LDL‐treated HAECs (Figure 4D, E). Besides, tube formation test results revealed that overexpressing LincRNA‐p21 facilitated the ability of tube formation in ox‐LDL‐treated HAECs, while the ability of tube formation was inhibited by further addition of miR‐221 mimic (Figure 4F).

### 3.5 miR‐221 inhibits SIRT1 expression to repress proliferation, migration and tube formation of HAECs

**FIGURE 5:**
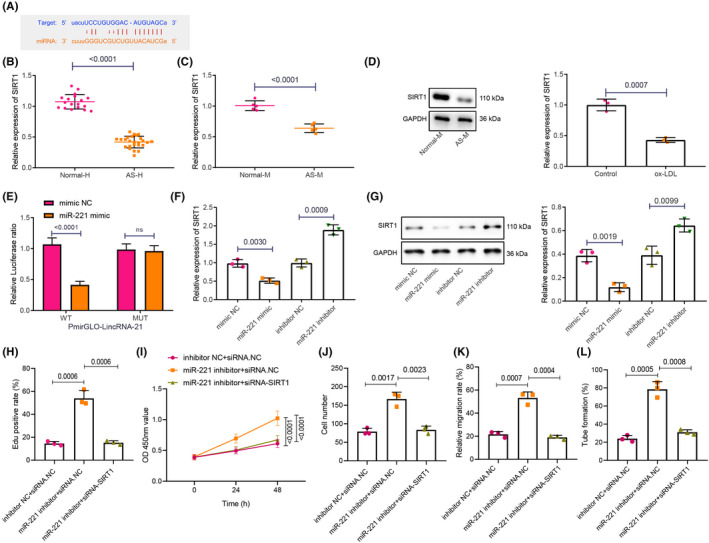
miR‐221 suppresses the SIRT1 expression in order to enhance cell proliferation, migration and tube formation of HAECs. A, The binding site between SIRT1 and miR‐221 was predicted by bioformatics website. B, SIRT1 expression in peripheral blood from patients with AS (n = 25) and normal patients (n = 18) was measured using RT‐qPCR. C, The mRNA expression of SIRT1 in AS tissues was detected using RT‐qPCR. D, The mRNA expression of SIRT1 in ox‐LDL‐treated HAECs was measured using RT‐qPCR. E, The relationship between SIRT1 and miR‐221 was verified using dual luciferase reporter gene assay. F, The mRNA expression of SIRT1 in HAECs was measured using RT‐qPCR. G, The mRNA expression of SIRT1 in HAECs was measured using western blot analysis. H, HAEC proliferation ability was assessed using EdU in response to miR‐221 inhibitor +siRNA‐SIRT1. I, HAEC proliferation ability was examined using CCK‐8. J, HAEC migrative ability was evaluated using Transwell assay. K, Wound healing ability in HAECs was monitored by the means of scratch test. L, Angiogenic ability in HAECs was tested using tube formation test. The experiment was repeated three times

To further study the downstream regulatory mechanism of miR‐221, we used a bioinformatics website (http://starbase.sysu.edu.cn/) and determined that SIRT1 might be a downstream target of miR‐221 (Figure 5A). We subsequently detected SIRT1 in human peripheral blood, mouse model and ox‐LDL‐treated HAECs with the help of RT‐qPCR, which showed that SIRT1 was poorly expressed in AS (Figure 5B‐D). Dual luciferase reporter gene assay verified that miR‐221 could reduce the luciferase activity of SIRT1, which suggested miR‐221 may have the potential to bind to SIRT1 (Figure 5E). RT‐qPCR and western blot analysis showed that overexpressing miR‐221 contributed to decreased SIRT1 expression in HAECs, whereas inhibiting miR‐221 resulted in increased SIRT1 expression (Figure 5F, G).

We then silenced SIRT1 in the presence of miR‐221 inhibitor, and performed EdU and CCK‐8 assays, which revealed that inhibiting miR‐221 promoted HAEC proliferation following ox‐LDL treatment, which was reversed by the further treatment of si‐SIRT1 (Figure 5H, I). Transwell assay, scratch test and tube formation test indicated that inhibiting miR‐221 had promoted the migrative and tube formation abilities of HAECs exposed to ox‐LDL, while silencing SIRT1 blocked the inhibition of miR‐221 on cell migrative and tube formation abilities in ox‐LDL‐treated HAECs (Figure 5J‐L).

### 3.6 miR‐221 inhibits the deacetylation of Pcsk9 by targeting SIRT1 and inhibits HAEC proliferation, migration and tube formation

**FIGURE 6:**
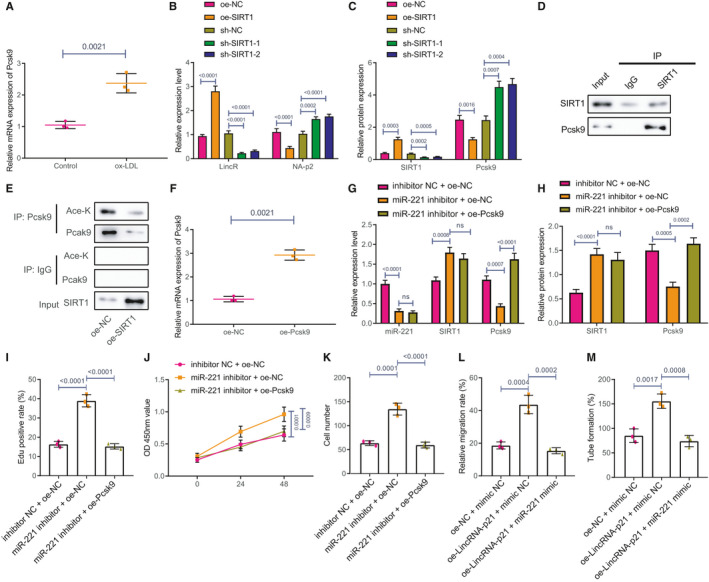
miR‐221 inhibits the deacetylation of Pcsk9 to suppress HAEC proliferation, migration, and tube formation through targeting and inhibiting the expression of SIRT1. HAECs were transfected with SIRT1‐mimic and SIRT1‐inhibitor, or co‐transfected with miR‐221 inhibitor +oe‐NC or miR‐221 inhibitor +oe‐Pcsk9. A, The mRNA expression of Pcsk9 in ox‐LDL‐treated HAECs was detected using RT‐qPCR. B, The mRNA expressions of Pcsk9 and SIRT1 in ox‐LDL‐treated HAECs were detected using RT‐qPCR. C, The protein expressions of Pcsk9 and SIRT1 were detected using western blot analysis. D, The interaction between SIRT1 and Pcsk9 in HAEC was detected by Co‐IP. E, After overexpressing the expression of SIRT1, the interaction between SIRT1 and Pcsk9 and the acetylation level were detected by Co‐IP. F, Pcsk9 expression was measured using RT‐qPCR. G, The expressions of miR‐221, SIRT1 and Pcsk9 were evaluated using RT‐qPCR. H, The protein expressions of SIRT1 and Pcsk9 were detected using western blot analysis. I, The proliferation of HAECs was detected using EdU. J, HAEC proliferation was examined using CCK‐8. K, HAEC migration was measured using Transwell assay. L, Wound healing ability was detected using scratch test. M, HAEC angiogenesis was detected using tube formation test. The experiment was repeated for 3 times

RT‐qPCR showed that Pcsk9 expression was increased in HAECs exposed to ox‐LDL (Figure 6A). Afterwards, HAECs were transfected with SIRT1‐mimic and SIRT1‐inhibitor, and the expressions of SIRT1 and Pcsk9 were detected using RT‐qPCR and western blot analysis, which suggested that Pcsk9 expression was notably decreased in SIRT1‐mimic‐transfected HAECs, whereas Pcsk9 expression was significantly increased in SIRT1‐inhibitor‐transfected HAECs (Figure 6B, C). The results of Co‐IP showed that in HAECs, SIRT1 was enriched in Pcsk9 gene (Figure 6D), and after overexpressing SIRT1, the correlation between SIRT1 and Pcsk9 was decreased and the level of acetylation was reduced (Figure 6E).

The efficacy of overexpressing Pcsk9 in HAEC was confirmed by RT‐qPCR (Figure 6F). Next, HAECs were co‐transfected with miR‐221 inhibitor +oe‐NC or miR‐221 inhibitor +oe‐Pcsk9. RT‐qPCR showed that silencing miR‐221 contributed to downregulated levels of miR‐221 and Pcsk9 but upregulated SIRT1 expression. However, the co‐treatment with miR‐221 inhibitor +oe‐Pcsk9 only resulted in an increased expression of Pcsk9 (Figure 6G). Similar results were obtained using western blot analysis (Figure 6H). Subsequently, CCK‐8 and EdU results showed that silencing miR‐221 promoted HAEC proliferation while overexpressing Pcsk9 inhibited the growth of HAECs (Figure 6I, J). Meanwhile, Transwell assay and scratch test results indicated that silencing miR‐221 promoted HAEC migration but the co‐treatment of miR‐221 inhibitor +oe‐Pcsk9 inhibited HAEC migration (Figure 6K, L). Moreover, tube formation test revealed that silencing miR‐221 promoted tube formation while the addition of oe‐Pcsk9 inhibited tube formation (Figure 6M).

### 3.7 Overexpression of LincRNA‐p21 inhibits endothelial cells apoptosis and the development of ApoE^−/−^ mice *via* mediating miR‐221/SIRT1/Pcsk9 axis

**FIGURE 7:**
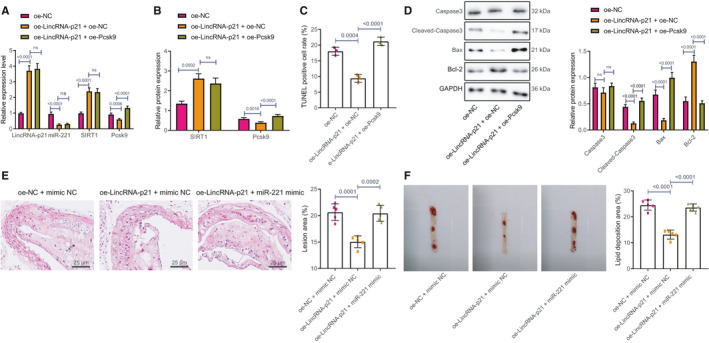
Overexpression of LincRNA‐p21 inhibits endothelial cell apoptosis and the development of ApoE^−/−^ mice *via* mediating miR‐221/SIRT1/Pcsk9 axis. A, LincRNA‐p21, SIRT1, miR‐221 and Pcsk9 expressions in plaque area were detected using RT‐qPCR. B, The protein expressions of SIRT1 and Pcsk9 were detected using RT‐qPCR. C, Apoptosis of carotid endothelial cells in mice was detected using TUNEL staining. D, The protein expressions of apoptosis‐related factors Cleaved‐Caspase‐3, Caspase‐3, Bax and Bcl‐2 were examined using western blot analysis. E, Carotid artery injury in mice was examined using HE staining. F, The plaque area of carotid artery in mice was detected by oil red O staining. n = 15. The experiment was repeated three times

The AS mice models were injected with adenovirus expressing oe‐LincRNA‐p21 + oe‐NC, or oe‐LincRNA‐p21 + oe‐Pcsk9. According to RT‐qPCR, overexpressing LincRNA‐p21 significantly increased the expressions of LincRNA‐p21 and SIRT1 but decreased miR‐221 and Pcsk9 expression. However, co‐treatment of oe‐LincRNA‐p21 + oe‐Pcsk9 only increased the expression of Pcsk9 (Figure 7A), as confirmed by western blot analysis (Figure 7B).

Apoptosis of carotid endothelial cells in mice was detected using TUNEL staining, which showed that overexpressing LincRNA‐p21 noticeably reduced the number of TUNEL positive cells, but the outcome was reversed by further treatment of oe‐Pcsk9 (Figure 7C). Moreover, western blot analysis suggested that overexpressing LincRNA‐p21 inhibited Cleaved‐Caspase‐3 and Bax expression but upregulated Bcl‐2 expression. Additionally, co‐treatment of oe‐LincRNA‐p21 + oe‐Pcsk9 contributed to elevated Cleaved‐Caspase‐3 and Bax expression but downregulated Bcl‐2 expression (Figure 7D).

HE staining showed that overexpressing LincRNA‐p21 contributed to significantly reduced plaque area, whereas co‐treatment of oe‐LincRNA‐p21 + oe‐Pcsk9 increased the plaque area (Figure 7E). Oil red O staining showed that overexpressing LincRNA‐p21 resulted in declined lipid deposition area but the co‐treatment of oe‐LincRNA‐p21 + oe‐Pcsk9 contributed to increased lipid deposition area (Figure 7F).

## 4 DISCUSSION

AS is a complex chronic disease characterized by the deposition of lipids on the walls of blood vessels, which can lead to the proliferation of smooth muscle, endothelial cells and immune cells.^20^ A previous study has revealed that LincRNAs can act as endogenous RNA sponges for miRNAs that can further alter the expression of miRNAs and their interplay in regulating the progression of AS.^7^ Therefore, this study demonstrated that overexpressing LincRNA‐p21 inhibited the development of AS in mice *via* mediating miR‐221/SIRT1/Pcsk9 axis.

Initially, miR‐221 expression was upregulated in patients with AS and in the AS mouse model. The expression of miR‐221 was notably elevated in an AS rat model and cultured endothelial cells.^21^ Besides, miR‐221‐3p expression was also validated to be strikingly upregulated in atherosclerotic vessels.^22^ We also found that miR‐221 inhibited HAEC proliferation, migration and tube formation in vitro; the findings of which can be supported by a previously reported article on the effects of overexpressing miR‐221‐3p in HAECs causing the accumulation of intracellular reactive oxygen species, thus further leading to cell apoptosis.^23^ Subsequent experiments revealed that LincRNA‐P21 acted as sponge for miR‐221 and LincRNA‐p21 and showed a negative correlation between LincRNA‐p21 and miR‐221 through Pearson correlation analysis. The relation between LincRNA‐p21 and miR‐221 has previously been validated. For example, LincRNA‐p21 upregulated FOS levels to sponge miR‐221, inhibiting the apoptosis of hippocampal neurons in streptozotocin‐diabetic mice.^24^

Secondly, we found that upregulating LincRNA‐p21 promoted HAEC proliferation, migration and tube formation through suppressing the expression of miR‐221. The expression of LincRNA‐p21 was substantially lower in the aortic plaques of ApoE^‐/‐^ mice in comparison with that in control mice, whereas LincRNA‐p21 inhibited cell proliferation and induced apoptosis of vascular smooth muscle cells and mouse mononuclear macrophages in vitro.^9^ Furthermore, miR‐221 was highly expressed in diabetic mice, whereas LincRNA p21 was poorly expressed. Overexpressing LincRNA p21 suppressed the apoptosis of hippocampal neuron in diabetic mice by sponging miR‐221.^24^ miR‐221 was demonstrated to target and inhibit the expression of SIRT1. Similarly, SIRT1 was confirmed as a target gene of miR‐221 and miR‐221 inhibited the proliferation and fibrosis‐related proteins by targeting SIRT1.^25^ Indeed, miR‐221 was negatively correlated with SIRT1 mRNA ^26^ and we also found that the expression of SIRT1 was decreased in AS. Related research showed that deficiency of SIRT1 promoted the development of AS.^27^ The expression of SIRT1 in ox‐LDL‐treated human umbilical vein vessel endothelial cells was decreased.^28^ The experiments showed that miR‐221 inhibited the deacetylation of Pcsk9 by targeting SIRT1 and inhibiting HAEC proliferation, migration and tube formation. Gain‐of‐function of SIRT1 activation reduced plasma LDL‐C levels through inhibiting Pcsk9 secretion.^29^ SIRT1 activator SRT3025 provided AS protection in Apoe^‐/‐^ mice by reducing hepatic Pcsk9 secretion.^30^ Moreover, the expression of Pcsk9 in AS was significantly higher and Pcsk9 knockdown inhibited ox‐LDL‐induced inflammatory response.^16^ Lastly, the in vivo experiments further validated that LincRNA‐p21 significantly reduced the plaque area in mice, inhibited Cleaved‐Caspase‐3 and Bax expression but upregulated Bcl‐2 expression, which inhibited the development of aortic AS. Conversely, plaque stability was characterized by an increased plaque collagen content and thickened fibrous cap, highlighting the involvement of SIRT in the inflammatory pathways of diabetic atherosclerotic lesions modulated by incretin.^31^ Overexpressing LincRNA‐p21 contributed to decreased expressions of Cleaved caspase‐3 and Bax but upregulated the expression of Bcl‐2.^24^

## 5 CONCLUSIONS

**FIGURE 8:**
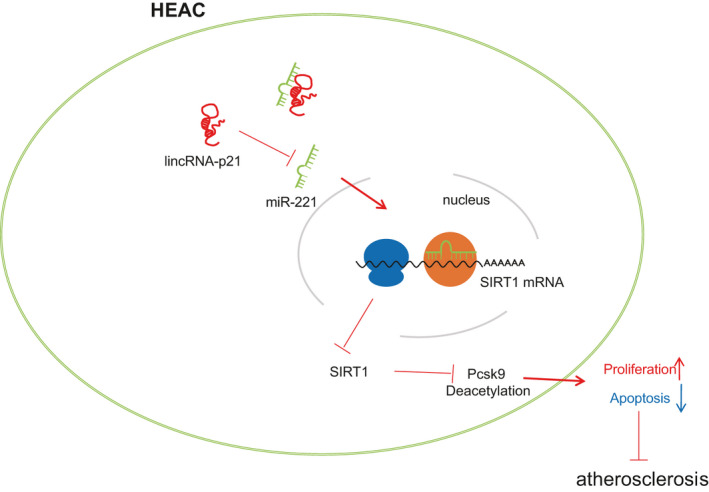
Diagram illustrating the molecular mechanism of how LincRNA‐p21 attenuates the progression of aortic AS *via* the miR‐221/SIRT1/Pcsk9 axis

In conclusion, elevated LincRNA‐p21 expression enhanced the promoter deacetylation of SIRT1 on Pcsk9 to protect against development of AS by binding to miR‐221 (Figure 8). These results draw attention to a potential molecular mechanism of LincRNA‐p21 in the treatment of AS. As stated by a prior study, increased inflammation is involved with the adipose tissue hypo‐expression of SIRT1, which exerts local and systemic effects and affect the cardiac performance in overweight patients with pre‐diabetes.^32^ However, this effect was not evaluated in the study population given the limited time and funding, thus warranting further investigations in the future.

## CONFLICTS OF INTEREST

The authors declare no conflicts of interest.

## AUTHOR CONTRIBUTIONS

**Haojie Wang:** Conceptualization (supporting); Writing‐original draft (supporting); Writing‐review & editing (supporting). **Fei He:** Data curation (supporting); Formal analysis (supporting); Funding acquisition (supporting); Writing‐original draft (supporting); Writing‐review & editing (supporting). **Bing Liang:** Data curation (supporting); Formal analysis (supporting); Funding acquisition (supporting); Writing‐original draft (supporting); Writing‐review & editing (supporting). **Yuanhu Jing:** Data curation (supporting); Formal analysis (supporting); Funding acquisition (supporting); Writing‐original draft (supporting); Writing‐review & editing (supporting). **Pei Zhang:** Data curation (supporting); Formal analysis (supporting); Funding acquisition (supporting); Writing‐original draft (supporting); Writing‐review & editing (supporting). **Weichao Liu:** Investigation (supporting); Writing‐original draft (supporting); Writing‐review & editing (supporting). **Bowen Zhu:** Conceptualization (supporting); Writing‐original draft (supporting); Writing‐review & editing (supporting). **Dongmei Dou:** Conceptualization (lead); Writing‐original draft (lead); Writing‐review & editing (lead).

## DATA AVAILABILITY STATEMENT

The datasets generated/analysed during the current study are available.

## Supporting information

Table S1

Supplementary Material
