# Supplementary material for: LincRNA‐p21 alleviates atherosclerosis progression through regulating the miR‐221/SIRT1/Pcsk9 axis
Source: J Cell Mol Med. 2021 Sep 19;25(19):9141–53. doi: 10.1111/jcmm.16771 (PMC8500963; doi:10.1111/jcmm.16771)
Supplement: Supplementary file 1 — Table S1 [file JCMM-25-9141-s002.docx]

**Supplementary Table 1** Primer sequences for RT-qPCR

| Gene | Primer sequences |
| --- | --- |
| hsa-LincRNA-p21 | F: 5´-GGGTGGCTCACTCTTCTGGC-3´ |
|  | R: 5´-TGGCCTTGCCCGGGCTTGTC-3´ |
| mmu-LincRNA-p21 | F: 5´-CCTGTCCACTCGCTTTC-3´ |
|  | R: 5´-GGAACTGGAGACGGAATGTC-3´ |
| hsa-miR-221 | F: 5´-ACCTGGCATACAATGTAG-3´ |
|  | R: 5´-GAACATGTCTGCGTATCTC-3´ |
| mmu-miR-221 | F: 5´-GCTACATTGTCTGCTGGG-3´ |
|  | R: 5´-GAACATGTCTGCGTATCTC-3´ |
| hsa-SIRT1 | F: 5´-TAGACACGCTGGAACAGGTTGC-3´ |
|  | R: 5´-CTCCTCGTACAGCTTCACAGTC-3´ |
| mmu-SIRT1 | F: 5´-GGAGCAGATTAGTAAGCGGCTTG-3´ |
|  | R: 5´-GTTACTGCCACAGGAACTAGAGG-3´ |
| hsa-Pcsk9 | F: 5´-CCTGAACTCTGCACCAAGTCCT-3´ |
|  | R: 5´-TCATCTGGCTCAGATAGGAGGG-3´ |
| mmu-Pcsk9 | F: 5´-CCTGAACTCTGCACCAAGTCCT-3´ |
|  | R: 5´-TCATCTGGCTCAGATAGGAGGG-3´ |
| hsa-U6 | F: 5´-GGGCAGGAAGAGGGCCTA-3´ |
|  | R: 5´-GAACATGTCTGCGTATCTC-3´ |
| mmu-U6 | F: 5´-CAGCACAAAAGGAAACTCACC-3´ |
|  | R: 5´-GCCTTGACAACTCATCTGAGCG-3´ |
| hsa-GAPDH | F: 5´-GTGGACCTGACCTGCCGTCT-3′ |
|  | R: 5´-GGAGGAGTGGGTGTCGCTGT-3′ |
| mmu-GAPDH | F: 5´-CTCGCTTCGGCAGCACA-3′ |
|  | R: 5´-AACGCTTCACGAATTTGCGT-3′ |

Note: hsa-LincRNA-p21, hsa-long intergenic non-coding RNA p21; mmu-LincRNA-p21, mmu-long intergenic non-coding RNA p21; hsa-SIRT1, hsa-sirtuin 1; mmu-SIRT1, mmu-sirtuin 1; hsa-Pcsk9, hsa-proprotein convertase subtilisin/kexin type 9; mmu-Pcsk9, mmu-proprotein convertase subtilisin/kexin type 9; hsa-U6, small nuclear RNA; hsa-GAPDH, hsa-glyceraldehyde-3-phosphate dehydrogenase
